# Chronic insulin treatment of diabetes does not fully normalize alterations in the retinal transcriptome

**DOI:** 10.1186/1755-8794-4-40

**Published:** 2011-05-15

**Authors:** Georgina V Bixler, Heather D VanGuilder, Robert M Brucklacher, Scot R Kimball, Sarah K Bronson, Willard M Freeman

**Affiliations:** 1 Department of Pharmacology, Penn State College of Medicine, 500 University Drive, Hershey, PA, USA; 2 Genome Sciences Facility, Penn State College of Medicine, 500 University Drive, Hershey, PA, USA; 3 Department of Cellular & Molecular Physiology, Penn State College of Medicine, 500 University Drive, Hershey, PA, USA

## Abstract

**Background:**

Diabetic retinopathy (DR) is a leading cause of blindness in working age adults. Approximately 95% of patients with Type 1 diabetes develop some degree of retinopathy within 25 years of diagnosis despite normalization of blood glucose by insulin therapy. The goal of this study was to identify molecular changes in the rodent retina induced by diabetes that are not normalized by insulin replacement and restoration of euglycemia.

**Methods:**

The retina transcriptome (22,523 genes and transcript variants) was examined after three months of streptozotocin-induced diabetes in male Sprague Dawley rats with and without insulin replacement for the later one and a half months of diabetes. Selected gene expression changes were confirmed by qPCR, and also examined in independent control and diabetic rats at a one month time-point.

**Results:**

Transcriptomic alterations in response to diabetes (1376 probes) were clustered according to insulin responsiveness. More than half (57%) of diabetes-induced mRNA changes (789 probes) observed at three months were fully normalized to control levels with insulin therapy, while 37% of probes (514) were only partially normalized. A small set of genes (5%, 65 probes) was significantly dysregulated in the insulin-treated diabetic rats. qPCR confirmation of findings and examination of a one month time point allowed genes to be further categorized as prevented or rescued with insulin therapy. A subset of genes (Ccr5, Jak3, Litaf) was confirmed at the level of protein expression, with protein levels recapitulating changes in mRNA expression.

**Conclusions:**

These results provide the first genome-wide examination of the effects of insulin therapy on retinal gene expression changes with diabetes. While insulin clearly normalizes the majority of genes dysregulated in response to diabetes, a number of genes related to inflammatory processes, microvascular integrity, and neuronal function are still altered in expression in euglycemic diabetic rats. Gene expression changes not rescued or prevented by insulin treatment may be critical to the pathogenesis of diabetic retinopathy, as it occurs in diabetic patients receiving insulin replacement, and are prototypical of metabolic memory.

## Background

Impairment or loss of vision due to diabetic retinopathy (DR) is one of the most debilitating complications of diabetes. Type 1 diabetes leads to some degree of diabetic retinopathy (DR) in approximately 95% of patients within 20 years of diagnosis [1,2], and the incidence of DR continues to increase due to the dramatic rise in the prevalence of Type 2 diabetes. Prevention by glycemic control through insulin replacement [3] and ablative laser surgery for advanced disease continue to be the standard of care. Currently there are no approved pharmacotherapeutic approaches for prevention and/or reversal of DR.

The Diabetic Control and Complications Trial (DCCT) demonstrated a significant reduction, but not elimination, of the development of diabetic complications in Type 1 diabetics through intensive insulin therapy as opposed to conventional insulin therapy [3]. The continuation study, Epidemiology of Diabetic Interventions and Complications (EDIC), revealed that patients formerly on conventional insulin therapy retained a higher risk for development of complications, including DR, even four [4] and ten [5] years after switching to intensive insulin therapy despite achieving equivalent glycosylated hemoglobin (HbA1c levels). This clinical phenomenon is also apparent with Type 2 diabetics as demonstrated by the United Kingdom Prospective Diabetes Study (UKPDS) where a "sustained legacy" of microvascular complication development was evident in the conventional treatment group despite being under intensive treatment recommendations for the past ten years [6]. As many Type I and II diabetes patients experience significant glycemic insults prior to diagnosis and/or do not achieve tight blood glucose control for a period of time after diagnosis, these studies suggest that these patients will be at a higher risk for complications development in the future even under intensive insulin therapy.

Animal models of diabetes demonstrate a similar phenomenon of DR development even with insulin therapy as the longitudinal clinical studies. Twenty years ago, retinopathy, as measured by retinal capillary aneurysms and lesions, was observed in dogs after 4.5 years of diabetes, despite good glycemic control for the last 2.5 years [7]. In rodent models of diabetes, islet cell transplantation after 6 weeks of diabetes, but not after 12 weeks of diabetes, reduced the development of DR when both groups were examined at 26 total weeks of diabetes [8]. A number of additional studies have examined this phenomenon at the level of retinal expression of specific genes and proteins in rodent models [9-11]. For example, in a Lewis rat STZ model, increased retinal expression of IL-1B, TNFα, ICAM-1 and iNOS with diabetes has been demonstrated to be prevented by good glycemic control, but once established can not be reversed by long-term insulin therapy [9]. In total, the clinical and basic research literature clearly supports the hypothesis that some retinal molecular alterations induced during a period of poor or no control cannot be reversed by subsequent good glycemic control and that other molecular alterations are not prevented from developing after initiation of insulin replacement. This phenomenon has been termed 'metabolic memory' [12]. The mechanisms through which metabolic memory is retained and the role of metabolic memory in DR disease development remain to be described [13]. The present study sought to address this hypothesis of persistent dysregulation of gene expression for the first time with whole genome analysis of retinal transcriptome with untreated diabetes and with insulin therapy.

## Methods

### Animal Methods

Animal studies were conducted using protocols approved by the Penn State Institutional Animal Care and Use Committee. Male Sprague-Dawley rats (Charles River, Wilmington, MA) were imported at 100-125 g body weights. After one week, rats were fasted overnight and diabetes was induced by intraperitoneal injection of 65 mg/kg streptozotocin (STZ; Sigma Aldrich, St. Louis, MO) in 10 mM sodium citrate vehicle, pH 4.5 as previously described [14-16]. Control rats received the same treatment, but were injected with an equal dose of vehicle only. Throughout the study, rats were maintained on a 12 hour light/dark cycle and had free access to food and water. To confirm induction of diabetes, blood glucose and body weight of all animals were assessed one week post-injection, and then biweekly throughout the study. Only STZ-injected rats with blood glucose levels >250 mg/dL at initial and subsequent testing were included in the diabetic groups.

Rats in the insulin treatment group received one 26 mg subcutaneous insulin pellet (LinShin Canada, Scarborough, Canada) delivered via trocar six weeks after induction of diabetes. An additional 26 mg implant was introduced when midday non-fasting blood glucose exceeded 250 mg/dL or when body weight exceeded 300 g. Control and untreated diabetic rats were treated similarly but received no insulin implants. Retinas for the insulin-treatment experiment (insulin-treated diabetic, untreated diabetic, and age-matched non-diabetic controls) were harvested at three months post-injection. A second set of rats (control and untreated diabetic groups only) were sacrificed one month after injection for use in the qPCR experiments.

At sacrifice, body weights were recorded and blood glucose concentrations and percent glycosylated hemoglobin were measured from tail-nick blood samples using a Lifescan glucose meter and Siemens DCA analyzer, respectively. Rats received a lethal 100 mg/kg dose of pentobarbital (Nembutal; Ovation Pharmaceuticals Inc., Deerfield, IL) by intraperitoneal injection. Corneas were hemisected and the lens and vitreous were removed. Whole retinas, including RPE, were rapidly excised with fine forceps and snap-frozen in liquid nitrogen for subsequent transcriptomic analysis. Throughout the study, all rats were maintained by the Penn State JDRF Animal Models Core in accordance with the Institutional Animal Care and Use Committee guidelines under specific pathogen-free conditions and monitored by quarterly sentinel testing.

### RNA isolation

Retinas were homogenized in 500 μL TriReagent (Molecular Research Center, Cincinnati, OH) by bead mill (Retsch TissueLyzer II, Qiagen, Valencia, CA) using 3 mm stainless steel balls at 30 Hz for 30 seconds, as previously described [14,16]. RNA was isolated from retinal homogenates using standard TriReagent/BCP disruption and phase separation. Following overnight precipitation by incubation with isopropanol at -20°C, RNA was purified using the Qiagen RNeasy Mini kit (Qiagen). Quantity and quality assessments were made by spectrometry (NanoDrop ND1000; Thermo Scientific, Wilmington, DE) and microfluidics chip (Agilent 2100 Expert Bioanalyzer Nano Chip, Agilent, Palo Alto, CA), respectively. Only samples with RNA integrity numbers >8 were used in analyses.

### Microarray analysis

Retinal transcriptomic analysis of insulin-treated diabetic, untreated diabetic, and age-matched non-diabetic rats (n = 8/group, three-month experiment) was performed using Illumina RatRef-12 microarray (Illumina, San Diego, CA) according to standard methods and as previously described [17,18]. First-strand cDNA was synthesized by reverse transcription from 500 ng input RNA by two-hour incubation at 42°C with T7 Oligo(dT) primer, 10 × First Strand buffer, dNTPs, RNase inhibitor, and ArrayScript. Second-strand cDNA was synthesized from first-strand cDNA by two hour incubation at 16°C with 10 × Second Strand buffer, dNTPs, DNA polymerase, and RNase H, purified using the Illumina TotalPrep kit (Ambion, Foster City, CA) according to manufacturer's protocols and eluted in 18 μL 55°C nuclease-free water. cRNA was synthesized from second-strand cDNA using the MEGAscript kit (Ambion), and labelled by incubation for 14 hours at 37°C with T7 10 × Reaction buffer, T7 Enzyme mix, and Biotin-NTP mix. Following purification with the Illumina TotalPrep RNA Amplification kit (Ambion) according to manufacturer's instructions, cRNA yields were quantitated using a NanoDrop ND1000 spectrometer. Biotinylated cRNA (750 ng) was hybridized to RatRef-12 BeadChips by incubating for 20 hours at 58°C at a rocker speed of 5. After incubation, BeadChips were washed and streptavidin-Cy3 stained, dried by centrifugation at 275 × g for 4 minutes and scanned and digitized using a Bead Station Bead Array Reader.

After initial quality control of arrays, three arrays from the diabetic group and one from the insulin-treated diabetic group were eliminated for high background, low wash stringency or due to large blemishes on the array. Average normalization with background subtraction was performed in GenomeStudio software (Illumina) and flat files were imported into GeneSpring GX11 (Agilent) software for data analysis. Using detection p-values generated by GenomeStudio, probes were filtered for only those with present or marginal calls in 100% of the samples in at least one of the three experimental groups. This ensured that 1) transcripts not reliably detected in the experiment were excluded from statistical analysis, and 2) that genes potentially expressed in only one experimental animal group (i.e., in non-diabetic, diabetic, or insulin-treated diabetic rats only) were retained. Differential gene expression was determined using a combination of pair-wise statistical p-value (t-test, P < 0.05) and |fold-change| cutoff >1.2 fold between non-diabetic and diabetic groups according to standards in the field [19] and as previously described [17,18]. All of the genes differentially expressed between the non-diabetic and diabetic groups were then further classified by the expression in the insulin-treated diabetic group. Genes significantly altered with diabetes were classified as not normalized, partially normalized, normalized, or inverted by insulin replacement. The full microarray dataset is available in the Gene Expression Omnibus, accession# GSE24423. Network analysis of differentially-expressed genes not normalized with insulin treatment was performed using Ingenuity Pathway Analysis software (Ingenuity Systems, Redwood City, CA) to identify potential networks/pathways of interest among these genes. Gene Set Analysis [20] was performed using GeneSpring GX with minimum set size of 10 and a p-value of <0.05, and Benjamini Hochberg false discovery rate (FDR) correction. Comparison gene sets were downloaded from MSigDB (Broad Institute) for Gene Ontology (c5, 1454 gene sets) and motif gene sets (c3, 836 gene sets).

### Quantitative RT-PCR

qPCR analysis of targets of interest was performed as previously described, using TaqMan Assay-On-Demand (Applied Biosystems, Foster City, CA) gene-specific primers and probes and a 7900 HT Sequence Detection System (Applied Biosystems) [14,16]. Both one month (non-diabetic and diabetic) and three month (non-diabetic, diabetic, and insulin-treated diabetic) were analyzed by qPCR. Relative gene expression was calculated with SDS 2.2.2 software using the 2^-ΔΔCt ^analysis method with β-actin as an endogenous control. β-actin expression was determined to be unchanged between animal groups in a preliminary absolute quantitation experiment (data not shown). For a full list of primer/probe sets, see Additional File 1. Based on the qPCR results, genes were subdivided into "rescue" and "prevention" categories depending on whether a significant change in transcript was or was not evident at the one month time point, respectively.

### Immunoblot analysis

Soluble protein was isolated from retinas contralateral to those used in the three month qPCR analysis by homogenization in 250 μL detergent-based protein lysis buffer (2.5 mM HEPES, 1 mM EDTA, 100 mM NaCl, 1 mM dithiothreitol, 1% Tween 20, 1 mM Na_3_VO_4_, 2 protease inhibitor tablets/15 mL). Insoluble material was removed by centrifugation at 10,000 × g for 10 minutes at 4°C. Protein concentrations were determined by Pierce BCA assay. Protein samples (20 μg) were separated on Criterion 4-20% gradient gels (Bio-Rad, Hercules, CA) and transferred to PVDF membranes (GE Healthcare). Membranes were incubated with primary antibodies [CCR-5 (sc-17833), Jak3 (sc-6932), and Litaf (sc-166719) from Santa Cruz Biotechnology, Santa Cruz, CA] and HRP-conjugated anti-mouse IgG (ThermoFisher Scientific, Waltham, MA), anti-mouse IgA (Santa Cruz Biotechnology), or anti-rabbit IgG (GE Healthcare) secondary antibodies. Blots were developed on film (ThermoFisher Scientific) using enhanced chemiluminescence (Pierce, Rockville, IL) and images were analyzed by digital densitometry using ImageQuant TL software (GE Healthcare). Resultant immunoblot data was standardized to the corresponding whole-lane densitometric volume of the total protein stained gel as described previously [21].

### Statistical Analysis

Microarray data were filtered and analyzed as described above. For all qPCR experiments, the data for individual samples were normalized to the corresponding β-actin signal. Statistical analyses were performed using SigmaStat 3.5 software, with two-tailed t-tests for pairwise comparisons (i.e., one-month diabetic vs. non-diabetic) and one-way ANOVA with Student Newman Keuls post-hoc tests for multiple group comparisons (i.e., three-month diabetic vs. insulin-treated diabetic vs. non-diabetic). Statistical significance was determined by P < 0.05. Pearson correlations between gene expression levels and blood glucose or percent glycosylated HbA1c utilized a P < 0.05 to determine significance.

## Results

### Animal Results

**Figure 1:**
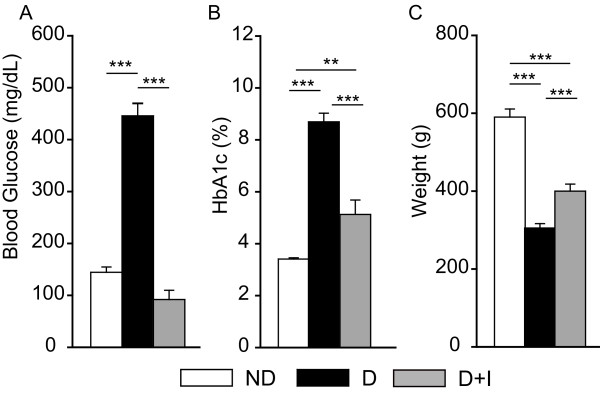
**Biometric data for experimental groups**. At sacrifice, blood glucose levels (A) were significantly higher in the diabetic rats than the control and insulin-treated groups. Similarly, glycosylated hemoglobin levels (B) were elevated in diabetic rats as compared to controls and insulin-treated diabetic rats. Diabetic rats were also underweight (C) as compared to controls and insulin-treated diabetic groups. One-way ANOVA, Student Newman Keuls pair-wise post-hoc test, **P < 0.01, ***P < 0.001, n = 7-10/group.

At sacrifice, diabetic rats were hyperglycemic compared to both non-diabetic rats and insulin-treated diabetic rats (Figure 1A). No differences at sacrifice in blood glucose were observed between non-diabetic and insulin-treated diabetic rats. Glycosylated hemoglobin levels were also elevated in diabetic rats (Figure 1B). Insulin-treated diabetic rats had significantly lower levels of glycosylated hemoglobin compared to diabetic rats but slightly elevated levels compared to non-diabetic rats. Rats in the diabetic group were underweight as compared to both non-diabetic and insulin-treated diabetic groups (Figure 1C). The mean weights of the insulin-treated diabetic rats were also less than those of the non-diabetic group.

At the one month timepoint used in the qPCR confirmation experiments, diabetic rats had significantly elevated blood glucose at sacrifice (321 ± 16 diabetic, 114 ± 12 non-diabetic, P < 0.001, t-test). The one month-diabetic rats were also underweight compared to non-diabetic controls (264 ± 16 diabetic, 391 ± 9 non-diabetic, P < 0.001). Glycosylated hemoglobin levels were not measured in the one month experiment.

### Transcriptome Profiling Results

**Figure 2:**
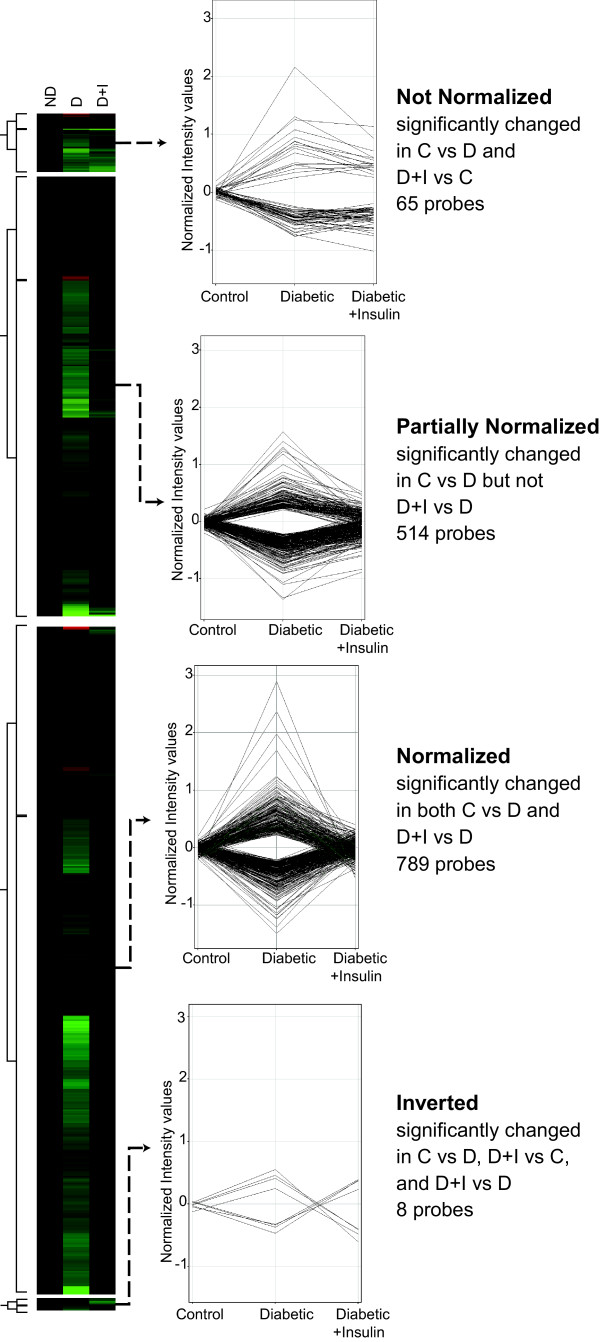
**Microarray analysis**. Retinal RNA from control (n = 8), diabetic (n = 5) and insulin-treated diabetic (n = 7) rats collected after the 3 months experimental paradigm were analyzed by Illumina Rat Ref12 microarray. 11,040 of 22,523 probes on the microarray had detectable signals. The 1,376 probes determined to be differentially expressed (P < 0.05, |fold change| >1.2) between control and diabetic animals were clustered according to expression in the insulin-treated diabetic group into Not Normalized, Partially Normalized, Normalized, and Inverted expression groups.

11,040 probes, out of the 22,523 probes on the arrays, passed the criteria for detectable expression. A total of 1,376 probes were determined to be differentially expressed between the non-diabetic and diabetic groups (P < 0.02, |fold change| >1.2). Probes differentially expressed between non-diabetic and diabetic groups were then clustered into Not Normalized, Partially Normalized, Normalized, and Inverted expression groups based on whether there was a significant difference in expression between non-diabetic and insulin-treated diabetic groups (Figure 2). Not Normalized probes (65, Additional file 2) were those for which there was a significant difference in the same direction between the non-diabetic group and both the diabetic and insulin-treated diabetic groups. Partially Normalized probes (514, Additional file 3) were those with a statistically significant change between the non-diabetic and diabetic groups that was sufficiently normalized for there to be no significant difference between the non-diabetic and insulin-treated diabetic comparison but not normalized to an extent where there was a significant difference between untreated diabetic and insulin-treated diabetic groups. Normalized probes (789, Additional file 4) were significantly regulated in the non-diabetic versus diabetic comparison and the diabetic versus insulin-treated diabetic comparison. A small number of probes (8, Additional file 5) displayed an Inverted profile in which the induction or reduction in the non-diabetic versus diabetic comparison flipped to a significant reduction or induction in the non-diabetic versus insulin-treated diabetic comparison.

**Figure 3:**
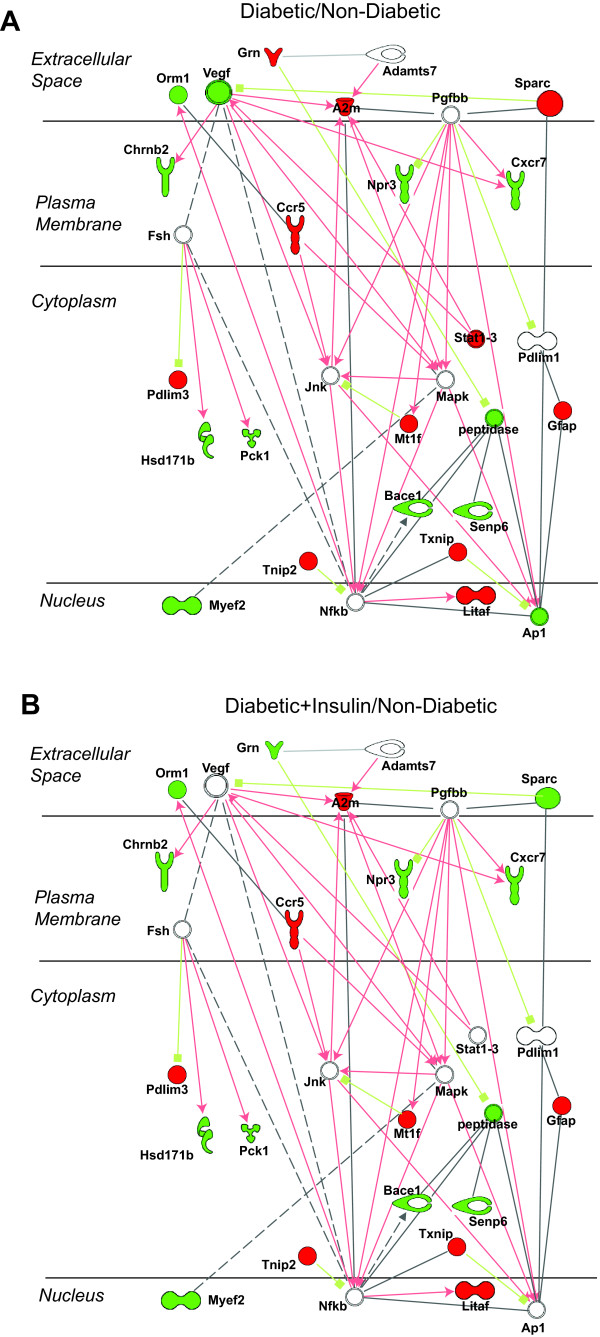
**Network analysis of genes Not Normalized**. Genes and expression values from the Not Normalized category were examined for networks of interrelated genes using Ingenuity Pathway Analysis software. One significant network was centered around Pdgf, Nfkb, and Jnk. Expression values for the control versus diabetic groups is presented in A and for the insulin-treated diabetic versus diabetic comparison in B. Relationships are presented as lines and arrows. Red lines represent activation or positive regulation of expression while green lines indicate inhibition or negative regulation of expression. Grey lines are known protein-protein interactions and dashed lines are indirect relationships. Gene symbols are coded as green for significantly reduced expression, red for significantly reduced expression, and white for no change in expression.

Analysis of gene networks extends the finding of sets of genes with differential patterns of expression in response to insulin therapy. For example, an inflammation related network of diabetes changes with nodes of Vegf, Pdgf, and NFkB was evident in the comparison of diabetics to non-diabetic controls (Figure 3A). This network was insensitive to insulin therapy (i.e., Not Normalized) (Figure 3B). In, contrast, a transcriptional network centred on Myc and altered in diabetic animals as compared to non-diabetic controls was almost completely normalized by insulin treatment (Additional File 6).

Gene Set Analysis was performed on all categories of changes, except for Inverted, for gene ontology and regulatory motif over-representation. AP2 binding sites and a CACCC motif with no known binding factor were enriched in the set of all changes with diabetes (P < 0.05, minimum of 5 genes). Gene Set Analysis of the Normalized subgroup identified Myc regulated genes as enriched, confirming the network analysis above. In the Partially Normalized subgrouping, a set of genes with the predicted mir-330 binding target (TGCTTTG) was over-represented, all of which were suppressed in diabetic and insulin-treated diabetic compared to non-diabetic controls. No enriched gene sets were found in the Not Normalized group.

Previously, we have examined whole-retina transcriptional changes in the same rat model after three months of diabetes using Illumina RatRef12 microarrays but with fewer samples per group (n = 4) and no insulin-treated diabetic group [18]. Comparison of the current dataset with the previous study identified 277 genes significantly regulated in the same manner as observed previously. Of the 27 genes selected for qPCR analysis 23 were observed in this previous study to be differentially regulated with diabetes in the microarray data. We have also previously reported a set of 14 retinal mRNA biomarkers for use in preclinical DR drug development studies [16]. Eleven of the 14 genes in the biomarker panel were identified as significantly regulated with diabetes in the current microarray analysis. The consistency of these expression changes across multiple independent studies suggests a robust and reproducible gene expression response in this model.

Extending these comparative analyses we examined the 77 transcripts previously reported by Gerhardinger and colleagues as differentially expressed in Müller cells isolated from Sprague Dawley rats six months after induction of diabetes by STZ injection [18]. Of the 63 genes that could be confidently cross-matched (based on Entrez Gene IDs) 17 were also significantly changed between non-diabetic and diabetic groups in this study. Importantly, all of these changes were in the same direction (up- or down-regulation) as the previous report. While the previous report specifically examined Müller cells isolated after 6 months of diabetes, the common changes suggest that the microarray analysis performed in this study of whole retinal tissue is able to detect changes occurring in the Müller cell population.

### Confirmation qPCR Results

An important element in characterizing retinal transcriptomic changes with diabetes that are unresponsive to insulin treatment is determining when these diabetes-induced changes first occur. In other words, do these changes occur before initiation of insulin treatment or after initiation of insulin treatment? To answer this question, specific target mRNAs from the whole genome gene expression analysis were also examined in separate non-diabetic and diabetic rat groups sacrificed one month after vehicle or STZ injection. Analysis of the data from the three month insulin intervention experiment in the context of this one month duration of diabetes experiment (i.e., prior to initiation of insulin treatment) permits further refinement of gene expression patterns into more specific Rescued and Prevented categories. Rescued, Partially Rescued, and Not Rescued are those gene expression changes which are manifest after one month of diabetes, before initiation of insulin treatment, and are normalized, partially normalized, or not normalized with chronic insulin treatment, respectively. Prevented, Partially Prevented, and Not Prevented groupings are those changes which have not occurred at one month of diabetes and do not occur by three months if insulin treatment is begun, occur by three months of diabetes at a significantly lesser magnitude with insulin treatment, or occur by three months of diabetes regardless of insulin treatment.

**Figure 4:**
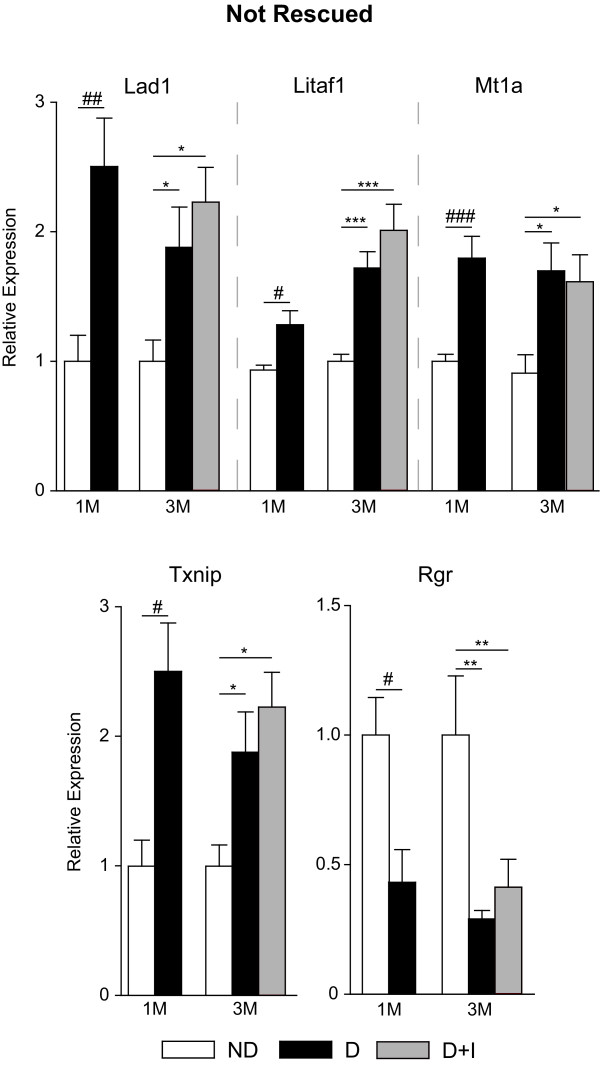
**Transcript expression not rescued by insulin treatment**. qPCR confirmation experiments also included 1 month control and diabetic groups to determine if retinal mRNA expression changes occurred before initiation of insulin treatment. Lad1, Litaf1, Mt1a, and Txnip all demonstrated a 'not rescued' molecular phenotype of increased expression in diabetic animals at 1 month of diabetes which remained elevated at 3 months in diabetic and insulin-treated diabetic groups. Similarly, Rgr expression was decreased at one month of diabetes and remained depressed at three months in diabetic animals with and without insulin treatment. One-way ANOVA, Student Newman Keuls pair-wise post-hoc test, *P < 0.05, **P < 0.01, ***P < 0.001 for 3 month data; t-test #P < 0.05, ##P < 0.01, ###P < 0.001 for 1 month data; n = 7-11/group for both time points. ND - Non-Diabetic control, D - Diabetic, D+I - Insulin-treated Diabetic, 1M - 1 month, 3M - 3 Months.

**Figure 5:**
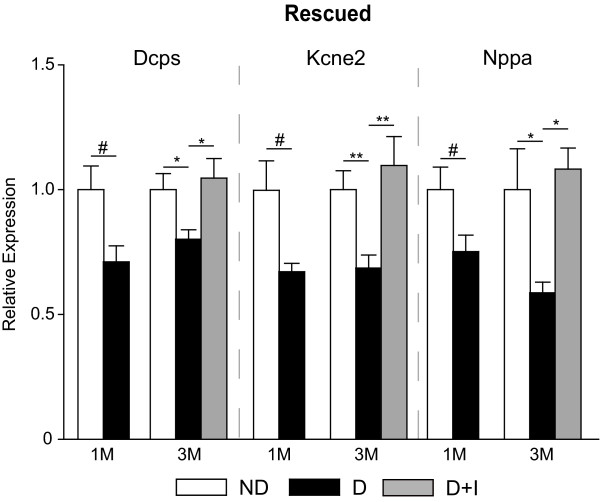
**Transcript expression rescued by insulin treatment**. Several transcripts were also confirmed by qPCR to demonstrate a 'rescued' phenotype. For these transcripts the altered mRNA expression in diabetic animals at one month was still observed in diabetic animals at three months but insulin-treatment normalized the expression. The decreased expression Dcps, Kcne2, and Nppa was brought to equivalent expression levels as controls at the three month timepoint by insulin treatment. One-way ANOVA, Student Newman Keuls pair-wise post-hoc test, *P < 0.05, **P < 0.01, ***P < 0.001 for 3 month data; t-test #P < 0.05, ##P < 0.01, ###P < 0.001 for 1 month data; n = 7-11/group for both time points. ND - Non-Diabetic control, D - Diabetic, D+I - Insulin-treated Diabetic, 1 M - 1 month, 3 M - 3 Months.

**Figure 6:**
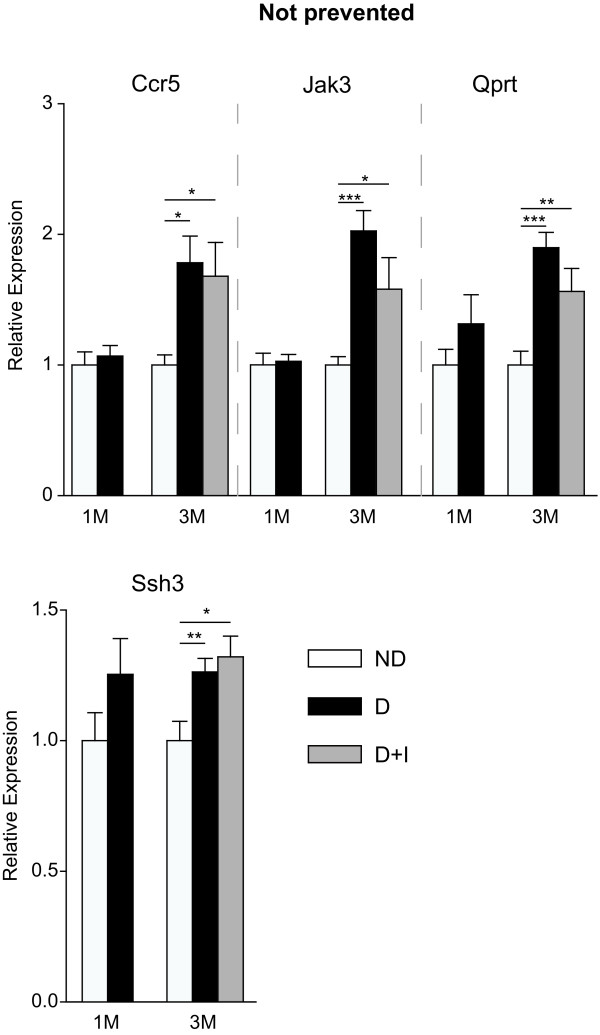
**Transcript dysregulation not prevented by insulin treatment**. In qPCR confirmation experiments, a set of genes was observed which were unaltered in expression after one month of diabetes but were differentially expressed at in both diabetic and insulin-treated diabetic groups at three months. This 'not prevented' phenotype was evident in the increased expression of Ccr5, Jak3, Qprt, and Ssh3 genes. One-way ANOVA, Student Newman Keuls pair-wise post-hoc test, *P < 0.05, **P < 0.01, ***P < 0.001 for 3 month data; n = 7-11/group. ND - Non-Diabetic control, D - Diabetic, D+I - Insulin-treated Diabetic, 1 M - 1 month, 3 M - 3 Months.

**Figure 7:**
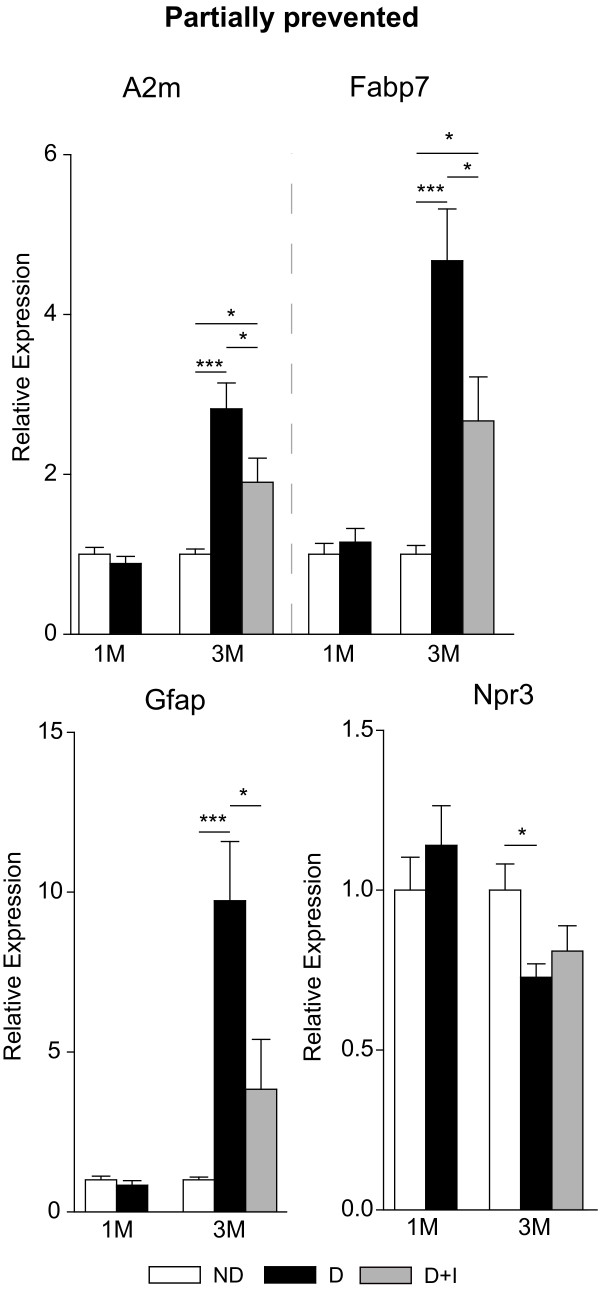
**Transcript dysregulation partially prevented by insulin treatment**. A set of gene in the qPCR experiments presented a 'partially prevented' phenotype in which gene expression changes in diabetic animals were significantly ameliorated by insulin treatment but altered expression from control animals was still evident. Increased expression of A2 m, Fabp7, and Gfap was reduced by insulin treatment but was still significantly higher than in the control group. Similarly, Npr3 expression in the insulin-treated diabetic rats was not significantly different from that in control rats, nor was it significantly different from the diabetic rats. One-way ANOVA, Student Newman Keuls pair-wise post-hoc test, *P < 0.05, ***P < 0.001 for 3 month data; n = 7-11/group. ND - Non-Diabetic control, D - Diabetic, D+I - Insulin-treated Diabetic, 1 M - 1 month, 3 M - 3 Months.

**Figure 8:**
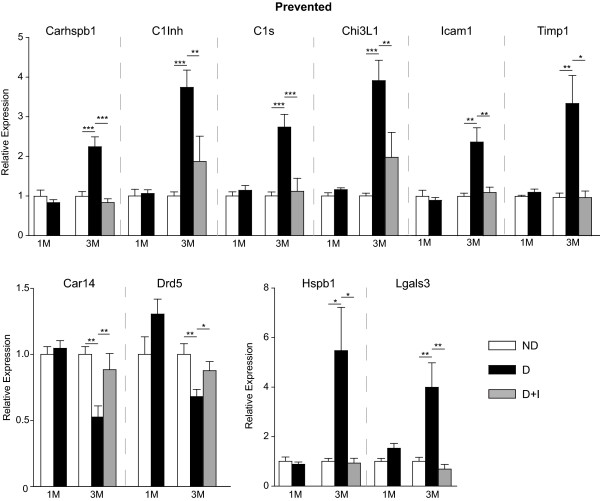
**Transcript dysregulation prevented by insulin treatment**. Genes which were confirmed in qPCR experiments to not be differentially expressed at one month but differentially expressed at three months in the diabetic but not the insulin-treated diabetic were classified as 'Prevented'. Increased expression of C1s, C1Inh, Carhspb1, Chi3L1, Hspb1, Icam1, Lgals3, and Timp1 was observed but completely prevented by one and a half months of insulin treatment. Similarly, decreased expression of Car14 and Drd5, was observed and prevented by insulin treatment. One-way ANOVA, Student Newman Keuls pair-wise post-hoc test, *P < 0.05, **P < 0.01, ***P < 0.001 for 3 month data; n = 7-11/group. ND - Non-Diabetic control, D - Diabetic, D+I - Insulin-treated Diabetic, 1 M - 1 month, 3 M - 3 Months.

A total of 27 targets from the microarray analysis were chosen for confirmation analysis including 15 from the Not Normalized, 5 from the Partially Normalized and 7 from the Normalized groups. While not comprehensive of all the genes observed with differential expression in the microarray analysis, the extensive qPCR confirmation experiments validate the quantitative accuracy of the discovery experiments. Twenty six of the 27 (96%) genes examined in this analysis were confirmed as being differentially regulated with diabetes in a statistically significant manner at the three month time-point (ANOVA, SNK post-hoc, P < 0.05). Eight of the 26 genes tested by qPCR were found to be differentially regulated after one month of diabetes. Using the qPCR data, genes were separated into different expression categories. Five were Not Rescued (Figure 4) and three were Rescued (Figure 5), with the remainder of genes dividing into four Not Prevented (Figure 6), four Partially Prevented (Figure 7), and 10 Prevented (Figure 8).

To assess whether the continued dysregulation of genes in the Not Rescued, Not Prevented and Partially Prevented gene categories was due to insufficient or unstable metabolic control, glycosylated hemoglobin levels at sacrifice were examined for correlation to gene expression in the insulin-treated diabetic animals. In the Not Prevented and Partially Prevented categories, only A2m, Ccr5, and Jak3 gene expression was significantly correlated to %HbA1c (Pearson correlation, P < 0.05), suggesting these genes may be responsive to small increases in %HbA1c such as those observed in the insulin-treated diabetic animals. None of the Not Rescued genes were correlated to the level of glycosylated hemoglobin in the insulin-treated diabetic animals, suggesting that these persistent changes are not related to blood glucose or %HbA1c levels in the time period immediately prior to sacrifice. However, other metabolic disturbances such as AGE-RAGE may account for the continuing dysregulation of these genes.

### Protein Expression Results

**Figure 9:**
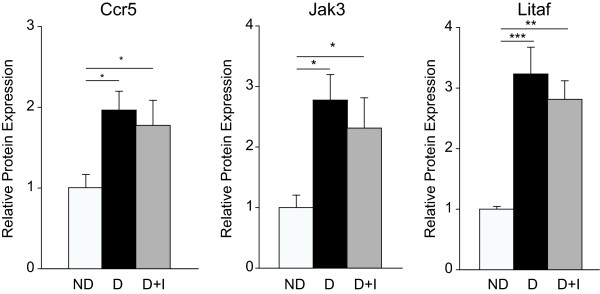
**Protein expression not normalized by insulin treatment**. Increased expression of Ccr5, Jak3, and Litaf1 was confirmed by immunoblotting. One-way ANOVA, Student Newman Keuls pair-wise post-hoc test, *P < 0.05, **P < 0.01, ***P < 0.001 for 3 month data; n = 5-7/group. ND - Non-Diabetic control, D - Diabetic, D+I - Insulin-treated Diabetic, 1 M - 1 month, 3 M - 3 Months.

While targeted analysis of the protein expression of every gene examined in this study is not feasible, confirmation that some of these changes in mRNA expression are reflected in altered protein expression is critical to the functional relevance of these alterations. Ccr5, Jak3, and Litaf protein expression was examined in the same three month animal cohort as the qPCR analysis, and was found to be significantly elevated in diabetic and insulin-treated diabetic groups compared to non-diabetic controls (Figure 9).

## Discussion

As DR occurs in Type I diabetes patients in spite of insulin therapy, this study sought to characterize the limitations of insulin replacement in normalizing diabetes-induced molecular changes in the retina by identifying gene expression alterations resistant to normalization by chronic insulin therapy. The use of a Type I diabetes animal model allows for the examination of retinal gene expression with and without insulin treatment and at different durations of diabetes in a manner not possible in humans.

### Gene expression alterations normalized with insulin treatment

As observed in the microarray analysis, the majority of diabetes-induced changes were normalized with insulin treatment. In confirmatory qPCR experiments that also examined a one month duration of diabetes, some of these normalized genes were further subdivided into "rescued" and "prevented" categories. The expression patterns of Kcne2, Nppa, and Dcps provide examples of how restoration of insulin and euglycemia can normalize diabetes-induced gene expression alterations that have occurred before initiation of therapy to non-diabetic levels. Additionally, a number of changes not evident at the one month time point were prevented from occurring with continued diabetes by insulin therapy. The majority of these genes examined in the confirmation experiments (i.e., Car13, Carhsbp1, Chi311, Timp1, C1inh, C1s, Hspb1, Icam1, Lgals3) are proinflammatory signalling molecules and have been described elsewhere [14,16,18,22,23]. These data provide clear evidence that animal model testing of novel DR treatments should be performed in the context of insulin replacement to more closely mimic the clinical scenario and assess the combinatorial effects of any potential therapies, as insulin therapy alone is insufficient to fully restore the normal retinal transcriptomic phenotype.

### Gene expression alterations not rescued by insulin therapy

In this study we identified a number of gene expression changes evident at 1 month of diabetes that were not reversed by chronic insulin therapy. The basement membrane anchoring filament protein Lad1 contributes to epithelial stability [24] and is regulated by both glucocorticoid receptor activation [25] and LPS stimulation [26]. Similarly, Mt1a is expressed in vascular components of the blood-retina barrier [27] and is regulated by pro-inflammatory signalling molecules, including factors released by Müller glia [28]. The regulation of these vascular function genes indicates a potential mechanism through which inflammation may contribute to vascular abnormalities in the diabetic retina. Several other inflammatory response genes induced with diabetes were also not rescued by insulin treatment. Litaf1, an inducer of TNFα expression [29], is a pro-inflammatory modulator induced in monocytes and macrophages in response to inflammatory stimuli such as LPS [30]. Txnip inhibits the antioxidant molecule thioredoxin, increases proinflammatory gene expression and signalling cascades [31]. Inhibition of Txnip in retinal endothelial cells prevents pathologies associated with early diabetic retinopathy (i.e., inflammation and gliosis) [32]. The induction of Lad1, Litaf1, Mt1a, and Txnip in the retina after 1 month of diabetes and the continued induction of these transcripts at three months of diabetes despite insulin replacement for the past 1.5 months suggest persistent alterations in glial and inflammatory processes, even with normalization of blood glucose levels. Rgr, a G protein-coupled receptor expressed almost exclusively in the retina [33] and localized to Müller glia and the retinal pigment epithelium [34], encodes a photoisomerase that contributes to pigment regeneration in photoreceptors. In Rgr-/- mice, dark adaptation is delayed and attenuated [35], suggesting that the suppression of Rgr observed could play a role in the well described deficits in dark adaptation in early DR. The expression pattern of these genes is consistent with the metabolic memory phenotype and demonstrates that even a one month period of loss of insulin signalling and hyperglycemia can result in molecular alterations that are unresponsive to insulin treatment. Recent work suggests that these metabolic memory prototypical changes may be regulated through epigenetic mechanisms [36,37].

### Diabetes-induced gene expression changes not prevented by insulin treatment

In addition to the gene expression changes at one month of diabetes Not Rescued by insulin treatment, the gene expression alterations identified in this study Not Prevented by insulin replacement and re-establishment of euglycemia are of importance for understanding DR in the context of insulin therapy and identifying targets for adjuvant therapies. The Not Prevented phenotype genes were expressed at non-diabetic control levels at one month of diabetes, but became significantly dysregulated with a longer duration (three months) of diabetes regardless of insulin treatment for the last one and a half months. Several proinflammatory genes exhibited this expression pattern. Ccr5, which mediates leukocyte attraction via chemokine receptor signalling, plays well characterized roles in inflammation [38]. In Type 1 and 2 diabetic patients, plasma Ccr5 is elevated and correlates to % glycosylated HbA1c levels [39,40]. We observed a similar positive correlation of CCR5 gene expression to glycosylated hemoglobin levels. Jak3, a proinflammatory gene expressed by immune cells and activated by interleukin receptors, is regulated by inflammation and tissue damage in the retina [41] in models of photic damage and retinal degeneration. We have previously reported the upregulation of Jak3 at three months of untreated diabetes in the STZ rat model [14,16] and the failure of insulin to prevent upregulation of Jak3 suggests that insulin therapy is insufficient to prevent some aspects of retinal inflammation with diabetes. Increased Qprt expression is also associated with tissue damage and neurodegeneration [42]. The protein product of this gene catalyzes the breakdown of neurotoxic quinolinate in the tryptophan nicotinamide adenine dinucleotide pathway, preventing the intracellular accumula tion of toxic compounds and maintaining neuronal function. By regulating actin/cofilin dynamics necessary for cytoskeletal plasticity, Ssh3 influences neuronal function and has been localized to the inner retinal layer in the eye [43], suggesting a role in ganglion cell function.

In addition to these gene expression changes, several others were only partially prevented by insulin therapy. A2m, a cytokine transporter induced by interleukin and predominantly expressed in blood vessel walls and the retinal pigment epithelium in the retina has also been described as systemically elevated in diabetes patients [44]. In retina of diabetic rodents, A2m expression increases in astrocytes and the inner limiting membrane, suggesting a broad inflammatory insult to both the neural and vascular retina [45]. Increased Gfap, a hallmark of gliosis, also increases with inflammation and diabetes [46-48]. This gene is expressed by astrocytes and Müller glia throughout the retina and likely contributes to inflammatory cascades and neurovascular dysfunction in diabetic retinopathy. Npr3, contributes to vascular regulation of electrolyte concentrations and fluid balance [49,50]. The failure of insulin to prevent dysregulated Npr3 expression may lead to an abnormal extracellular environment in the retina that has the potential to induce inflammation and neuronal dysfunction.

Together, the findings of this study demonstrate the ability of chronic insulin therapy, initiated after one and a half months of uncontrolled diabetes, to normalize the majority of retinal gene expression changes. However, chronic insulin therapy fails to completely restore the retinal transcriptome to a non-diabetic control phenotype. Additionally, at least some of these transcript changes are reflected in altered abundance of the corresponding proteins. Given the well supported hypothesis that a feed-forward cycle of inflammation and neurovascular dysfunction is a causative factor in DR development [51,52], it is not surprising that the majority of genes dysregulated with diabetes and not normalized by insulin are implicated in inflammatory responses and neuronal and vascular function. As such, these changes may represent the first stages of this feed forward cycle. Some previously reported pro-inflammatory molecules (e.g, IL-1β and TNFα) were not detectable, suggesting a low level of mRNA expression. Recent reports have emphasized the dependence of the duration of hyperglycemia on the induction of these factors [53]. Future studies will need to assess whether the observed induction of pro-inflammatory factors, such as Ccr5, Litaf, Txnip, and Jak3, result in further activation of prototypical inflammatory signals at a later time.

These results also agree with the clinical findings of past hyperglycemic periods continuing to confer an increased the risk of DR and other diabetic complications long after restoration of euglycemia [5]. Additional studies are needed to examine other complications for similar molecular profiles. This ability of insulin to normalize much, but not all, of the non-diabetic control transcriptomic phenotype may attenuate or delay the development of DR pathology, but ultimately the continued dysregulation of neuronal, vascular, and inflammation related genes may lead to more classical aspects DR development such as edema, leukostasis, and neuronal apoptosis.

The regulatory mechanisms underlying the retinal gene expression changes observed in this study remain to be determined. The nearly complete normalization of the majority of diabetes-induced changes (Rescued and Prevented categories) by insulin treatment points to their regulation by insulin signalling and/or hyperglycemia. Expression of genes in the Not Rescued category was not correlated to blood glucose or glycosylated hemoglobin and first occur after just one month of diabetes and before we have observed changes in retinal vascular permeability or apoptosis [14]. This indicates that the dysregulation of these genes is most likely not due to irreversible tissue damage. Previous reports suggest that many of the genes observed to be treatment resistant are localized to Müller, endothelial, and neuronal cell populations. While further studies are necessary to localize these changes to specific cell types, these results suggest that the observed changes are not the result of deposited leukocytes. An alternate mechanism for the un-responsiveness of these genes may be through epigenetic mechanisms such as altered chromatin status or differential DNA methylation of promoter regions [37,54]. Genes in the Not Prevented and Partially Prevented groupings are unchanged at 1 month of uncontrolled diabetes but are dysregulated at three months of diabetes despite blood glucose control for the prior 1.5 months. These dysregulations may occur, despite insulin therapy, as a secondary effect of the genes not rescued with insulin or due to the non-physiologically regulated release of insulin in these rats.

Future studies will need to examine the effects of intensive insulin therapy or beta cell transplantation on normalization of retinal gene expression. Ultimately, protein expression studies and localization of changes to specific cellular populations will help provide the necessary distinction between adaptive and pathological changes. We have already characterized a small number of insulin therapy resistant changes in retinal protein expression with diabetes [18] and these transcriptomic findings suggest that the quantitative proteomic analysis of retinal protein expression not normalized by insulin treatment is warranted. Individually, the genes discovered in this study to be not normalized by insulin treatment are targets for functional analyses using genetic and pharmacological approaches. Drug discovery efforts that target diabetes-related molecular alterations that are not normalized by insulin are needed to develop an adjuvant therapy that, in conjunction with insulin replacement, restores retinal gene expression and tissue function to a healthy state.

## Conclusions

The goal of the current study was to identify molecular alterations induced in the retina with diabetes that are not normalized by chronic insulin treatment, using a well-characterized rodent model of insulin-dependent diabetes. Transcriptomic analysis and extensive targeted confirmations demonstrated that, although insulin normalizes expression of the majority of genes altered after three months of untreated diabetes, expression of a number of genes remain partially or totally not normalized following therapy. This work demonstrated 1) that insulin alone is insufficient to completely return the diabetic retinal molecular phenotype to a non-diabetic control state, and 2) that the limitations of insulin therapy are manifested as both a failure to reverse some alterations that occur before the initiation of insulin therapy and an inability to prevent some changes that occur after the initiation of therapy.

## Competing interests

The authors declare that they have no competing interests.

## Authors' contributions

GVB conducted the microarray and qPCR assays and contributed to manuscript preparation. HDV performed informatic analyses and prepared the manuscript. RMB carried out the microarray data analysis. SRK assisted in the design of the experiment and contributed to the writing of the manuscript. SKB performed the animal experiments and contributed to the writing of the manuscript. WMF designed the study, prepared the figures and edited the manuscript. All authors read and approved the final manuscript.

## Pre-publication history

The pre-publication history for this paper can be accessed here:

http://www.biomedcentral.com/1755-8794/4/40/prepub

## Supplementary Material

Additional file 1 **Gene names and qPCR primers**. Full genes names, identifiers, aliases, and TaqMan probe numbers for genes examined in qPCR experiments. Click here for file

Additional file 2 **Not Normalized probes**. Microarray data for the probes in the Not Normalized cluster. Data provided includes the Illumina ProbeID, p-value for each pair-wise comparison, fold change and direction of regulation for each pairwise comparison, gene symbol, Entrez Gene ID#, and gene name. Click here for file

Additional file 3 **Partially Normalized probes**. Microarray data for the probes in the Partially Normalized cluster. Data provided includes the Illumina ProbeID, p-value for each pair-wise comparison, fold change and direction of regulation for each pairwise comparison, gene symbol, Entrez Gene ID#, and gene name. Click here for file

Additional file 4 **Normalized probes**. Microarray data for the probes in the Normalized cluster. Data provided includes the Illumina ProbeID, p-value for each pair-wise comparison, fold change and direction of regulation for each pairwise comparison, gene symbol, Entrez Gene ID#, and gene name. Click here for file

Additional file 5 **Inverted probes**. Microarray data for the probes in the Inverted cluster. Data provided includes the Illumina ProbeID, p-value for each pair-wise comparison, fold change and direction of regulation for each pairwise comparison, gene symbol, Entrez Gene ID#, and gene name. Click here for file

Additional file 6 **Normalized network**. Example gene network normalized with insulin treatment. A gene network centered around c-myc that was differentially expressed with diabetes was almost completely normalized by insulin treatment. Expression values for the control versus diabetic groups are presented in A and for the insulin-treated diabetic versus diabetic comparison in B. Relationships are presented as lines and arrows. Red lines represent activation or positive regulation of expression while green lines indicate inhibition or negative regulation of expression. Grey lines are known protein-protein interactions and dashed lines are indirect relationships. Gene symbols are coded as green for significantly reduced expression, red for significantly reduced expression, and white for no change in expression. Click here for file
