# Supplementary figures and images for: Chronic insulin treatment of diabetes does not fully normalize alterations in the retinal transcriptome
Source: BMC Med Genomics. 2011 May 15;4:40. doi: 10.1186/1755-8794-4-40 (PMC3113923; doi:10.1186/1755-8794-4-40)

**A**

Diabetic/Control

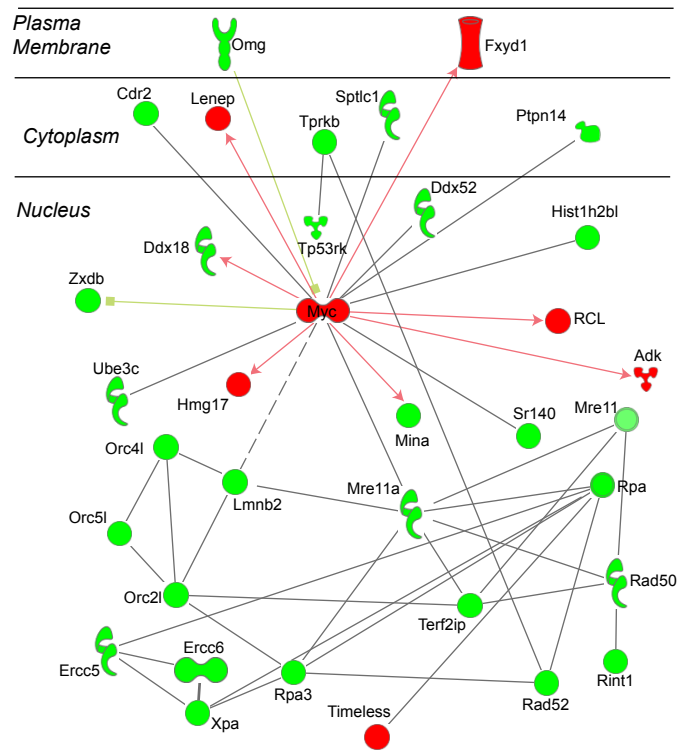**B**

Diabetic+Insulin/Control

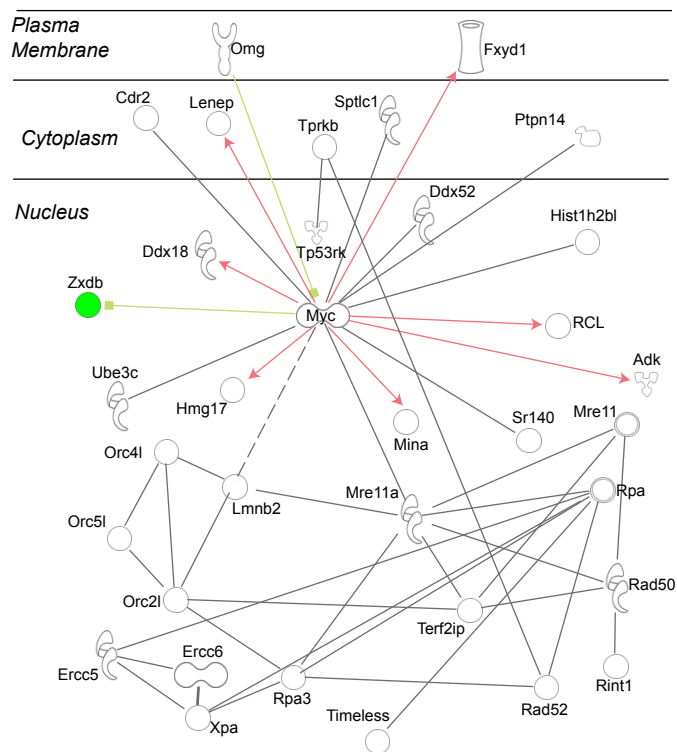

Supplement: Additional file 6 — Normalized network. Example gene network normalized with insulin treatment. A gene network centered around c-myc that was differentially expressed with diabetes was almost completely normalized by insulin treatment. Expression values for the control versus diabetic groups are presented in A and for the insulin-treated diabetic versus diabetic comparison in B. Relationships are presented as lines and arrows. Red lines represent activation or positive regulation of expression while green lines indicate inhibition or negative regulation of expression. Grey lines are known protein-protein interactions and dashed lines are indirect relationships. Gene symbols are coded as green for significantly reduced expression, red for significantly reduced expression, and white for no change in expression. [file 1755-8794-4-40-S6.PDF]
